# Disentangling the HIV-1 human protein interaction networks and their implications in the dynamics of viral replication and pathogenesis

**DOI:** 10.1186/1471-2334-14-S3-P61

**Published:** 2014-05-27

**Authors:** Devi Priyanka Maripuri, Daniel Alex Anand

**Affiliations:** 1 Department of Bioinformatics, Sathyabama University, Chennai-600119, India

## Background

Network biology has broadened our view and changed our perspective in understanding disease. A systems perspective is imperative to the understanding of the dynamics of viral pathogenesis and host interactions. Probing the complex web of HIV-1 - host protein interactions divulged biologically meaningful results.

## Methods

Global and protein specific networks were constructed using the HIV-1, Human Protein Interaction Database. The networks were visualized and analyzed using cytoscape and its plug ins. Functional annotation and enrichment analysis were performed using DAVID. Protein Sub cellular locations were determined using the LOCATE database.

## Results

Sixteen HIV-1 proteins interacted with 2545 human proteins, of which 3668 (34.53%) were direct and 6340 (65.46%), regulatory interactions. Seventy seven different types of interactions with T cell surface glycoprotein CD4 isoform 1 precursor (276/10008) and DNA dC->dU editing enzyme APOBEC-3G(109) being the top two interacting proteins. The top interacting HIV-1 proteins were *tat*, p14 (28%) followed by gp120 and *nef* p27. Functional annotation returned 312 clusters with highest enrichment scores of 30.71 for positive regulation of apoptosis (*p* value 7.9E-32). Toll like receptor and the Jak sTAT signaling pathways were most crucial to host response. LOCATE identified 491(10.7%) nuclear proteins, 419(9.15%) cytoplasmic, 295 (6.44%) membrane and 190(4.15%) extracellular proteins.

## Conclusion

Network theory and application is critical in understanding host - viral dynamics. Integration of the interaction networks, expression data, cell type, disease stage and other factors are bound to enable phenomenal insights that would accelerate the development of highly effective therapeutic interventions.

